# Quantitative analysis of change in bone volume 5 years after sinus floor elevation using plate-shaped bone substitutes: a prospective observational study

**DOI:** 10.1186/s40729-023-00501-2

**Published:** 2024-02-19

**Authors:** Kyoko Takafuji, Yutaro Oyamada, Wataru Hatakeyama, Hidemichi Kihara, Nobuko Shimazaki, Akihiro Fukutoku, Hiroaki Satoh, Hisatomo Kondo

**Affiliations:** 1 Department of Prosthodontics and Oral Implantology School of Dentistry, Iwate Medical University, 19-1 Uchimaru, Morioka, 020-8505 Japan; 2 Present Address: Department of Fixed Prosthodontics and Oral Implantology, Aichi Gakuin University, Nagoya, Japan

**Keywords:** Dental implants, Sinus floor elevation, Bone substitute, Bone volume

## Abstract

**Purpose:**

Tricalcium phosphate (TCP) has osteoconductive ability and reportedly offers similar clinical results as autogenous bone grafts in dental implant treatment. However, few reports quantify temporal changes in augmented bone volume after sinus augmentation. We aimed to establish a three-dimensional (3D) quantification method to assess bone volume after sinus augmentation and to evaluate biocompatibility of the TCP plate.

**Methods:**

Maxillary sinus floor augmentation was performed employing the lateral window technique, and plate-shaped β-TCP (TCP plate) was used instead of granular bone grafting materials. After lifting the sinus membrane, the TCP plate was inserted and supported by dental implants or micro-screws. The changes in bone volumes in the maxillary sinus before and after surgery were recorded using cone-beam computed tomography, saved as Digital Imaging and Communications in Medicine-formatted files, and transformed to Standard Triangle Language (STL)-formatted files. Pre- and post-operative STL data of bone volume were superimposed, and the augmented bone volume was calculated. Moreover, changes in bone volumes, TCP plate resorption rates, and bone heights surrounding the implants were three dimensionally quantified.

**Results:**

Fifteen implants in nine subjects were included in this study. TCP plates secured long-term space making, with results similar to those of granular bone substitutes. Newly formed bone was identified around the implant without bone graft material. TCP plate was absorbed and gradually disappeared.

**Conclusions:**

A novel 3D quantification method was established to evaluate changes in bone volume. Clinical application of TCP plate in sinus augmentation could be a better procedure in terms of prognosis and safety.

## Background

In most patients with severely atrophied maxillae, bone graft procedures are required for the placement of dental implants. Maxillary sinus floor augmentation, also termed as sinus augmentation, using lateral window technique was first reported by Tatum in 1976 [1]. Subsequently, several basic and clinical studies related to this procedure have been reported. In 1980, Boyne and James reported cases of maxillary sinus floor elevation using cancellous bone taken from the iliac crest [2].

Autogenous bone grafts have osteogenic, osteoinductive, and osteoconductive properties, and have been proven to be superior to artificial bone substitutes as they are slowly replaced by newly formed host bone [3–6]. However, several disadvantages are associated with autogenous bone grafts like: (i) requirement of a donor site (e.g., mandibular ramus in the oral cavity or iliac crest and tibia outside the oral cavity) which could result in post-operative pain and complications; (ii) morbidity from bone harvest; (iii) possibility of blood loss or hematomas, infections, injuries or cosmetic deformity at the explanation site; (iv) prolonged operative time; and (iv) invasive nature of the surgery [7].

Different types of artificial bone substitutes have been developed in the recent years, thereby increasing the frequency of bone grafts being performed without autogenous bone. Hydroxyapatite (HA), tricalcium phosphate (TCP) have been widely used as bone substitutes [8–11]. In general, HA has a very low resorption rate in the human body and can also slow new bone formation [12]. Thus, it reduces the bone-to-implant contact, and could potentially affect the prognosis of implants.

In contrast, TCP has been reported to have a high resorption rate in the human body [13]. TCP can be classified into alpha (α) or beta (β) phase, depending on its crystalline structure. α-TCP is formed at 1125℃ or higher, while β-TCP is formed between 900 and 1100℃ [14, 15]. β-TCP has osteoconductive ability, and is capable of gradual resorption, thus, providing space for bone regeneration [16, 17]. The efficacy of TCP has already been demonstrated in the field of orthopedics [18]. Several studies have used TCP for implant treatment and have reported results similar to autogenous bone grafts [19–23]. Studies using osteoblast-like and osteoprogenitor cells have demonstrated that the osteoconductive ability of β-TCP is capable of enhancing cell attachment and proliferation [24, 25]. Furthermore, TCP has been reported to progressively increase the expression of osteogenic proteins like alkaline phosphatase, osteonectin, osteopontin, and bone sialoprotein II [24]. This osteoconductivity of TCP is believed to induce osteoblasts, and enhance osteogenic proteins, thereby leading to new bone formation [26].

Sinus augmentation has been used, most frequently, as a bone augmentation technique for dental implant placement, and has a good prognosis regardless of the type of graft material used [27–31]. However, there have been only a few reports quantifying changes in augmented bone volumes after sinus augmentation. Furthermore, bone volume has largely been evaluated using two-dimensional panoramic radiographs [19, 32]; whereas, cone-beam computed tomography (CBCT) can be used not only for treatment planning, but also for three-dimensional (3D) quantitative evaluation of bone resorption around the implants.

Therefore, we hypothesized that it is possible to evaluate accurate bone volumes after sinus augmentation in 3D, applying digital technology. This study aimed to establish a novel evaluation method to three dimensionally quantify the changes in bone volume after sinus augmentation with TCP plates.

## Methods

### Patients

This study included patients who underwent sinus augmentation using TCP plates at our clinic between July 2013 and July 2014. Patients were considered eligible for inclusion in this study if they were aged at least 20 years, were healthy with good oral hygiene and motivation, had less than 5 mm of residual vertical height, and were scheduled to undergo sinus augmentation using TCP plates as the only graft material. There were no gender exclusion criteria.

Exclusion criteria included poor oral hygiene, diabetes [glycated hemoglobin (HbA1c) > 6.5%], other uncontrolled systemic diseases, and smoking habit. Patients who had undergone previous sinus augmentation within the past 5 years or if they simultaneously required other bone grafting techniques, such as guided bone regeneration or split crest were also excluded from the study. Furthermore, patients with intraoperative mucosa membrane perforation were excluded.

Informed consent was obtained from all patients for use of bone grafting materials and surgical procedures, as well as for multiple CBCT scans. The study was approved by the Ethics Committee of the Iwate Medical University (No. 01266). This study was registered with UMIN-CTR (UMIN000042854).

### Surgical procedure

**Fig. 1:**
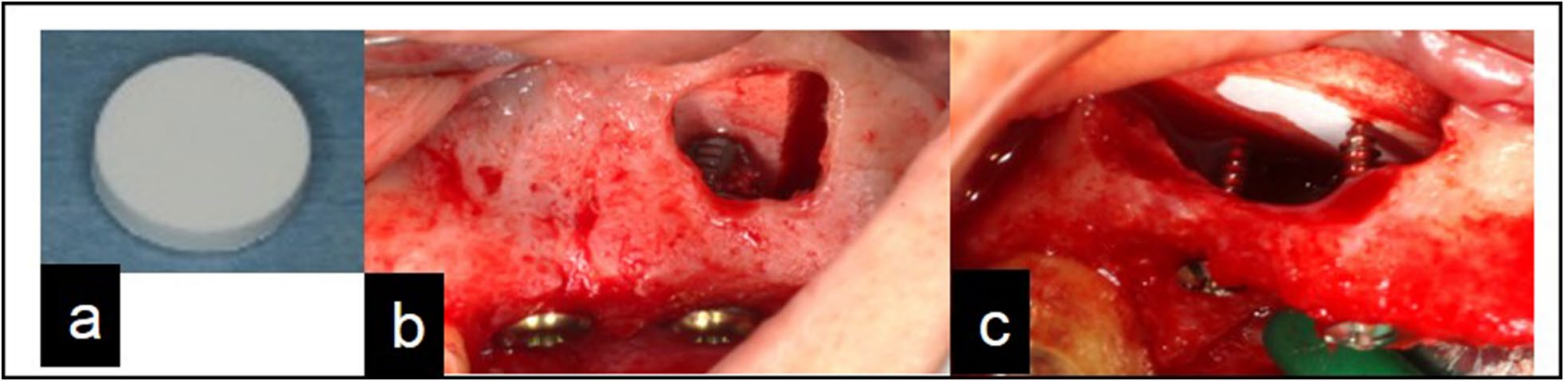
Surgical procedure. **a** Plate-shaped tricalcium phosphate (TCP). **b** TCP plate supported with implants. **c** TCP plate supported with micro-screws

All patients received oral hygiene instructions and oral prophylaxis before sinus augmentation. All the sinus augmentation procedures in this study were performed by two skilled specialists. The grafting material used was plate-shaped β-TCP [Ca_3_(PO_4_)_2_] (calcium phosphate) (Brain Base Corporation, Tokyo, Japan) (Fig. 1a), with a diameter of 10 mm and a thickness of 2 mm.

A bony window was prepared on the lateral wall of the sinus, and the sinus membrane was carefully removed and elevated. After lifting the sinus membrane, the TCP plate was inserted and supported by either dental implants (Nobel Biocare; Zürich-Flughafen, Switzerland, Straumann; Basel, Switzerland or GC; Tokyo, Japan) or micro-screws (ProSeed, Tokyo, Japan) (Fig. 1b, c). Further, a bioresorbable membrane (BIOMEND^®^, HAKUHO, Tokyo, Japan) was placed to cover the lateral window and to prevent soft tissue infiltration. All cases were performed according to the submerged surgical protocol for implant placement, and, in all cases, the number, diameter, and length of implants were determined using top-down treatment planning. In two cases, implant placement was performed 6 months after sinus augmentation, while in the other six cases, implants were placed simultaneously. The secondary surgery was performed 6 months after implant placement, and prosthetic treatment was done one month after the secondary surgery.

### Radiographic examination

To verify serial changes in the bone volume of the maxillary sinus, CBCT imaging (3D Accuitomo F17, Morita, Kyoto, Japan) was performed before surgery (T0) and at subsequent time points, including 1 year (T1), 2 years (T2), and 5 years (T3) after surgery. The acquisition parameters were as follows: current, 5 mA; exposure time, 17.5 s; field of view (FOV), 100 mm diameter × 100 mm height or FOV 100 mm diameter × 50 mm height; and isotropic pixel size, 0.25 mm. The images were reconstructed with a slice thickness of 1.0 mm.

The following measurements were performed by the two specialists certified by Japanese Society of Oral Implantology. Those two examiners discussed and decided the position of measurement points to find the real border, and to prevent the oversight on the X-ray images. Moreover, those who performed the surgery, did not touch the measurement process to avoid the subjective judgement.

### Augmented bone volume

**Fig. 2:**
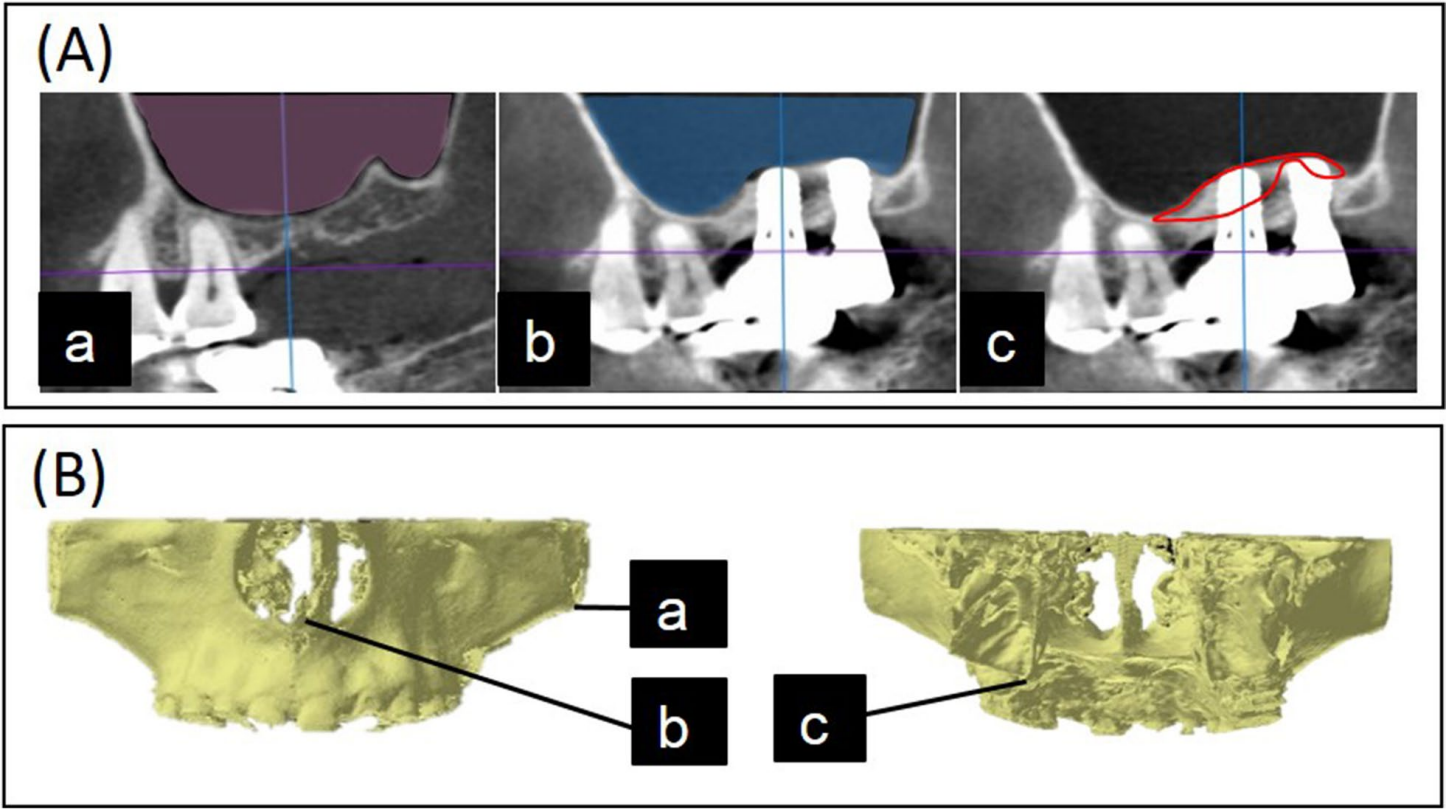
**A** Cone-beam computed tomography (CBCT) analysis. **a** An image of the maxillary sinus was extracted from the Digital Imaging and Communications in Medicine (DICOM) files of CBCT at T0. **b** An image of the maxillary sinus was extracted from the DICOM files of CBCT at T1, T2, or T3. **c** After deleting the overlapping part from before and after surgery, the remaining part (in the red line) was measured. **B** Reference points for superimposition. **a** The zygomatic process of maxilla. **b** The anterior nasal spine of maxilla. **c** The pterygoid hamulus of sphenoid bone

Maxillary sinus images were extracted from CBCT Digital Imaging and Communications in Medicine (DICOM) files at T0, T1, T2, and T3 (Fig. 2A) using image analysis software (OsiriX, Pixmeo SARL, Geneva, Switzerland).

Three-dimensional construction of maxillary sinus was performed using axial images of CBCT; ranging from the floor of maxillary sinus to the lower inferior nasal concha. Maxillary sinus was selected from all the consecutive slices in the above range. Subsequently, 3D maxillary sinus models were converted into Standard Triangle Language (STL) formatted files. The entire maxillae were extracted from the converted STL files since a reference point was required for superimposing 3D images of the sinus. Defects and halation due to X-ray artifacts of STLs were corrected using 3D scanning software (Geomagic Wrap, 3D SYSTEMS, Tokyo, Japan). STL files of maxilla and the maxillary sinus at T0 and T1, T0 and T2, and T0 and T3 were superimposed using the best-fit method by inspection software (spGauge, ARMONICOS, Shizuoka, Japan). The zygomatic process of maxilla, the anterior nasal spine of maxilla, and the pterygoid hamulus of sphenoid bone were used as the reference points for superimposition (Fig. 2B). Subsequently, STL data of maxilla and maxillary sinus before and after surgery were superimposed, and only the STL data of maxillary sinus were extracted. STL files that superimposed T0 and T1, T0 and T2, and T0 and T3 were then imported into reverse-engineering software (Geomagic Design X, 3D SYSTEMS, Tokyo, Japan). Boolean option was used to delete the overlapping sections of the 3D model before and after surgery, and the remaining part was considered the lift amount, with measurement of the resulting volumes (Fig. 2A). The lifted amount, representing augmented bone volume was quantified using Geomagic Design X. In addition, reduction rates of bone volume around the implants were calculated based on the difference between T1 to T2 and T1 to T3.

### Resorption rate of TCP plate

**Fig. 3:**
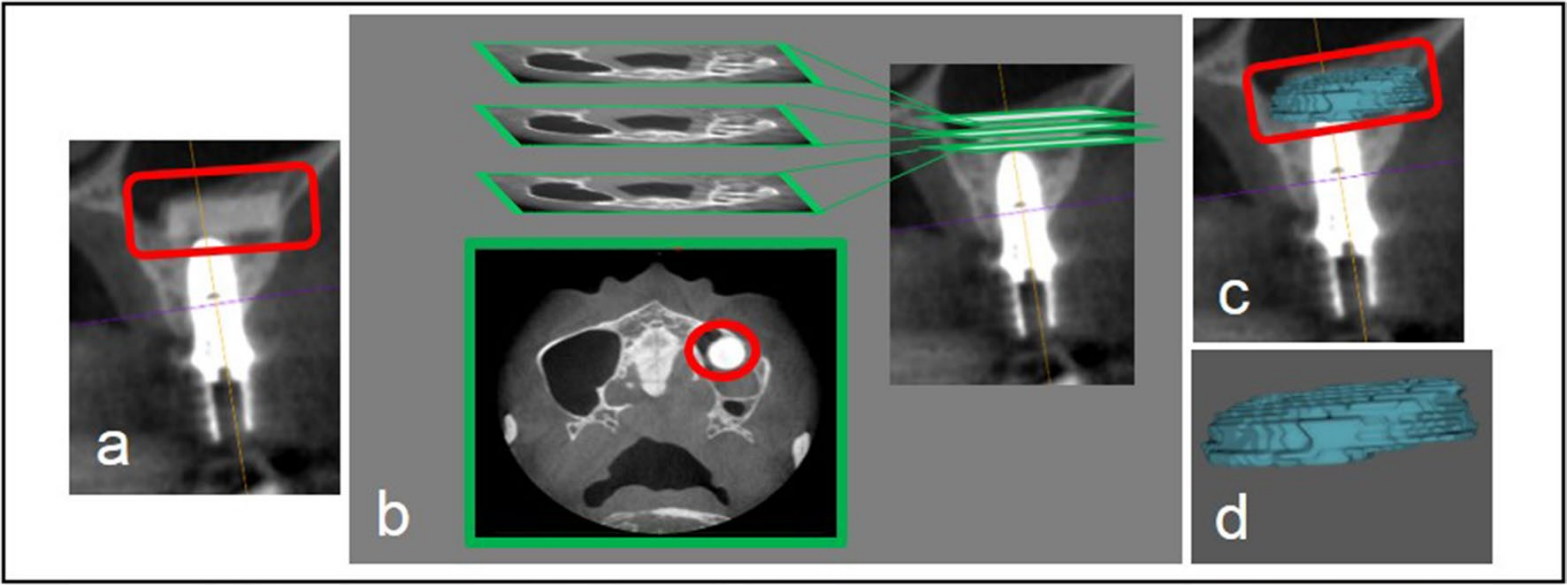
Three-dimensional construction of the TCP plate using axial images from CBCT.** A** CBCT data with TCP plate in the red frame. **b** Selection of the borders of the lesion on every slice of the sequence. **c, d** Creating 3D models of the TCP plate (the TCP plate is in the red frame)

TCP plate images were extracted from CBCT DICOM files at T1, T2, and T3 using image analysis software (OsiriX, Pixmeo SARL, Geneva, Switzerland) (Fig. 3). Volume of the 3D model of TCP plate was quantified by this software. The rate of decrease from T1 to T2 and from T1 to T3 was calculated as the resorption rate.

### Bone reduction rate surrounding the implants

**Fig. 4:**
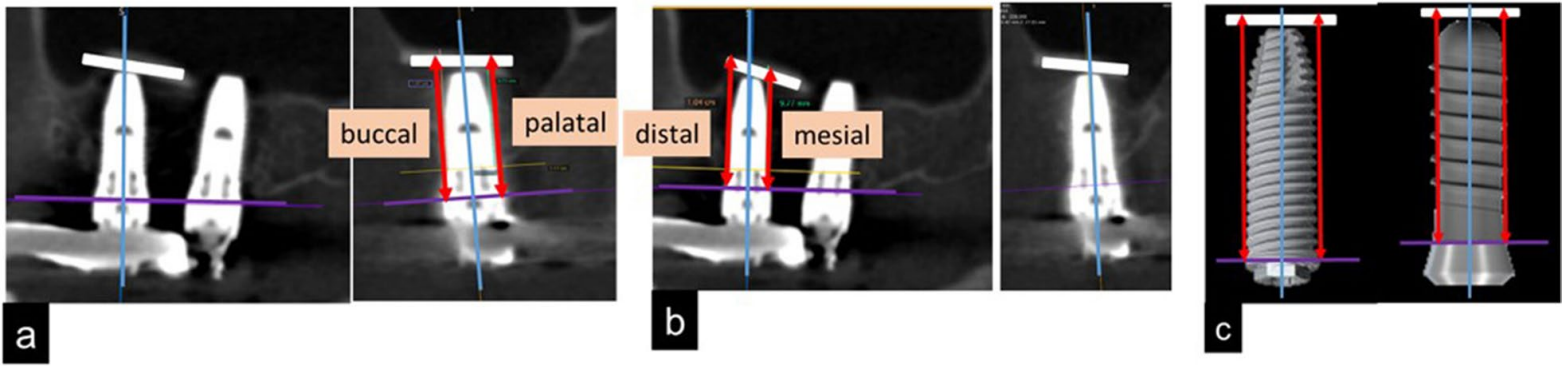
Measurement of bone height surrounding the implant. **a** Measurement of the buccal and palatal sides. **b** Measurement of the mesial and distal sides. **c** Measurement standard

Height of the bone surrounding the implant was measured at T1, T2, and T3 using image analysis software (OsiriX, Pixmeo SARL, Geneva, Switzerland). The mesial and distal sides were measured on the sagittal section, while the buccal and palatal sides were measured on the coronal section (Fig. 4). The distance from the platform to the floor of maxillary sinus, parallel to the long axis of implant body was denominated as the height of the bone (Fig. 4). This measurement was performed by an implant specialist with 20 years of clinical experience. Bone height reduction rates were calculated from T1 to T2 and from T1 to T3.

### Statistical analysis

We statistically analyzed bone volumes, resorption rates of TCP plates, and heights of bone surrounding implants. Since normal distributions could not be confirmed for any data from kurtosis and skewness, comparisons between the two groups were performed using Wilcoxon signed-rank test (ystat2004, Ishiyaku Publishers, Inc, Tokyo, Japan). A value of *p* < 0.05 was considered statistically significant.

## Results

### Subjects

A total of 15 implants (11 Nobel Biocare, 2 Straumann, and 2 GC) and nine TCP plates placed in the maxillary molar region in eight patients (5 females and 3 males), with an average age of 56.8 (range 45–64) years, were included in this study (Table 1).

**Table 1:** Characteristics of the study patients

Patients	Sex	Age	Missing region	TCP plate site	Implant site	Implant	Preoperative bone height (mm)	Thickness of the sinus membrane (mm)
1	Female	54	#3, #4	#3	#3	NSG φ4.0 × 10 mm	2	1
					#4	NSG φ4.0 × 10 mm	2	1
2	Female	45	#14	#14	#14	NSG φ4.0 × 10 mm	1	2
3	Female	62	#4, #5	#5	#4	GCs φ3.8 × 10 mm	1	0.3
					#5	GCs φ3.8 × 10 mm	4	0.4
4	Female	64	#2, #3	#3	#2	NSG φ4.0 × 11.5 mm	2	4
					#3	NSG φ4.0 × 11.5 mm	3	3
5	Male	64	#14, #15	#15	#14	NSG φ4.0 × 11.5 mm	3	1
					#15	NSG φ4.0 × 11.5 mm	4	1
6	Male	52	#2, #3	#3	#2	NSG φ4.0 × 10 mm	1	1
					#3	NSG φ4.0 × 10 mm	4	1
7	Female	58	#2, #3	#3	#2	St SP φ4.1 × 8 mm	2	1
					#3	St SP φ4.1 × 8 mm	3	1
8	Male	55	#12, #13, #14, #15	#14	#14	NSG φ4.0 × 11.5 mm	2	0.3
				#15	#15	NSG φ4.0 × 11.5 mm	1	0.2

*NSG* Nobel Speedy Groovy, *GCs* GC Setio Plus, *StSP* Straumann SP

Preoperative bone height: preoperative residual vertical bone height

Thickness of the sinus membrane: the thickness of the mucous membrane of maxillary sinus

### Clinical evaluation

**Fig. 5:**
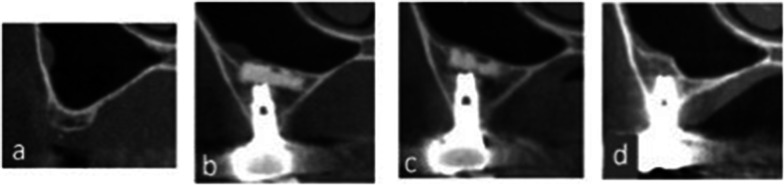
Representative frontal X-ray images of patient no. 3.** a** Before surgery. **b** 1 year after surgery. **c** 2 years after surgery. **d** 5 years after surgery

Implant placement and sinus augmentation were performed simultaneously in six cases, while a staged procedure was employed in the other two. There was no case of maxillary sinus mucosa membrane perforation. At the time of secondary surgery, all implants had successfully osseointegrated without any abnormalities in the maxillary sinus. The implant survival rate was 100% over the observation period of 5 years. X-ray computed tomography (CT) analysis revealed that TCP plates had remained in a stable position up to 2 years after surgery, thus, maintaining the space for new bone growth. Moreover, it was also observed that majority of the TCP plates had resorbed spontaneously within 5 years of surgery (Fig. 5).

### Image analysis

#### Augmented bone volume

The average augmented bone volume was 1491.8 mm^3^ after 1 year of surgery (T1), 1308.1 mm^3^ after 2 years (T2), and 1230.9 mm^3^ after 5 years (T3). The average reduction rate of augmented bone volume was 15% from T1 to T2 and 22% from T1 to T3.

However, increase in bone volume after sinus augmentation was observed in two cases at T2 and one case at T3 (Table 2).

**Table 2:** Reduction rates in augmented bone

Patients	Augmented bone volume (mm^3^)			Reduction rate of augmented bone volume (%)	
	T1	T2	T3	T1 → T2	T1 → T3
1	441.6304	355.7243	258.1486	19	42
2	1625.3948	1331.6264	1435.5207	18	12
3	1523.9913	880.8954	1411.5946	42	7
4	1360.2325	1350.1759	1059.9768	1	22
5	1358.0637	740.6942	256.3824	45	81
6	963.573	997.9204	938.7103	− 4	3
7	1843.0951	1565.8461	1579.4244	15	14
8	2818.6166	3241.6698	2907.7889	− 15	− 3
Average	1491.8247	1308.0691	1230.9433	15	22

T1, T2, and T3 indicate 1 year, 2 years, and 5 years after surgery, respectively

**Fig. 6:**
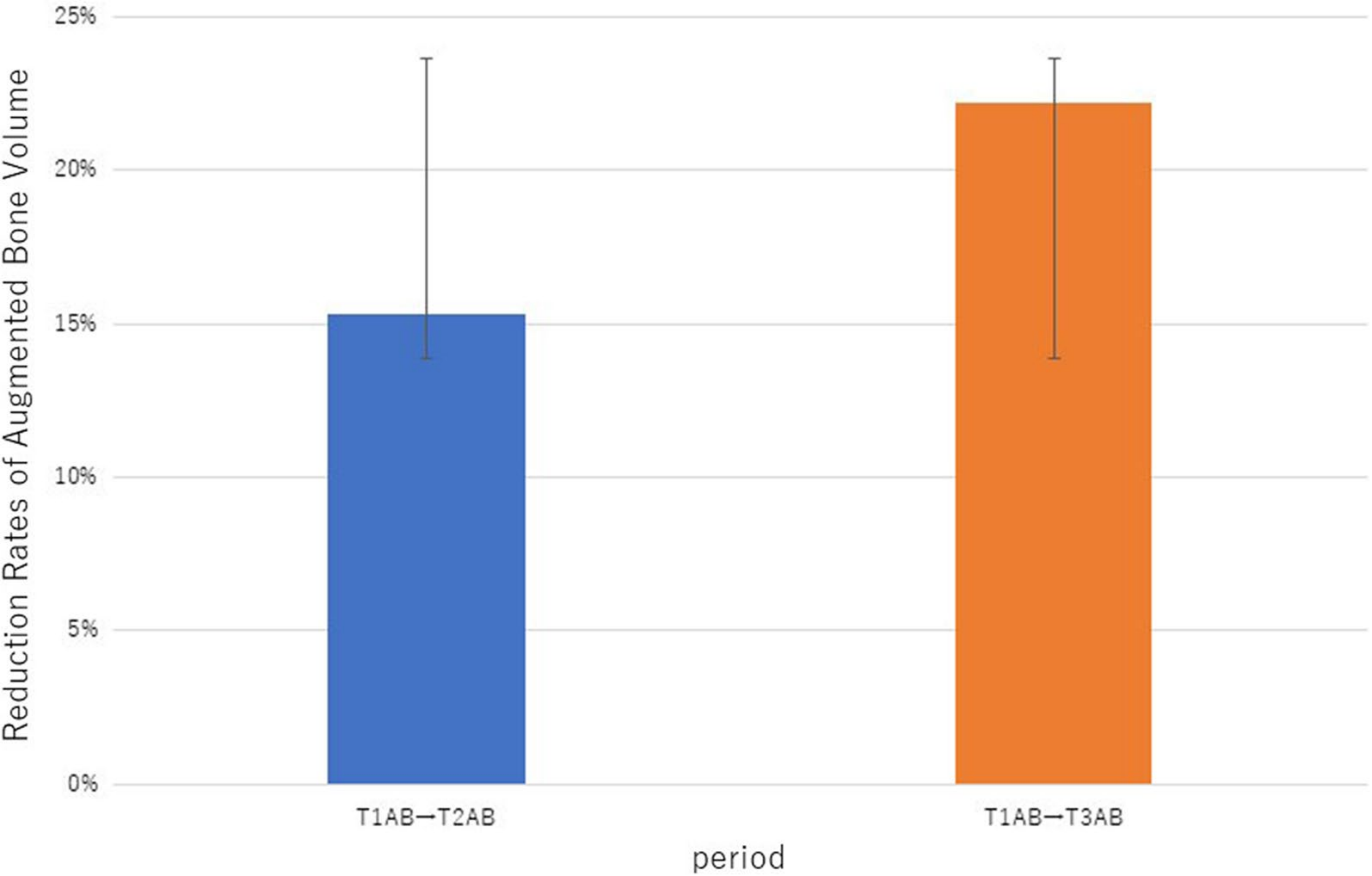
Reduction rates of augmented bone volume. T1, T2, and T3 indicate 1 year, 2 years, and 5 years after surgery, respectively

As shown in Fig. 6, the average reduction rate of augmented bone volume was 15% from T1 to T2, and 22% from T1 to T3. No significant difference was found in the average reduction rates of augmented bone volume between the two groups (*p* = 0.337) (Fig. 6)

#### Resorption rate of the TCP plates

**Fig. 7:**
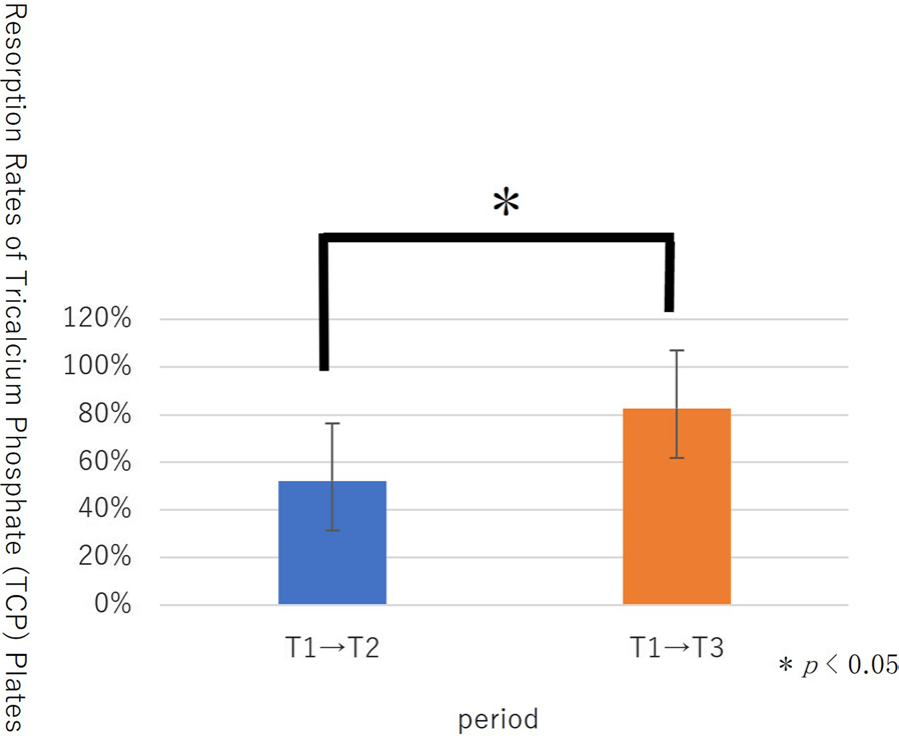
Resorption rates of tricalcium phosphate (TCP) plates

The average resorption rate of the TCP plates was 52% from T1 to T2, with a maximum resorption rate of 88% and a minimum of 18%. The average resorption rate of the TCP plates was 83% from T1 to T3, with maximum and minimum rates of 100% and 41%, respectively. The average resorption rate from T1 to T3 was significantly higher than T1 to T2 (*p* = 0.008) (Fig. 7).

#### Bone reduction rate surrounding implants

From T1 to T2, the average reduction rate in the bone height surrounding the 15 implants was 6% each on the buccal and palatal sides; and 5% each on the mesial and distal sides. Bone height increased on the buccal side in three cases, palatal side in two, mesial side in three, and distal side in five.

**Fig. 8:**
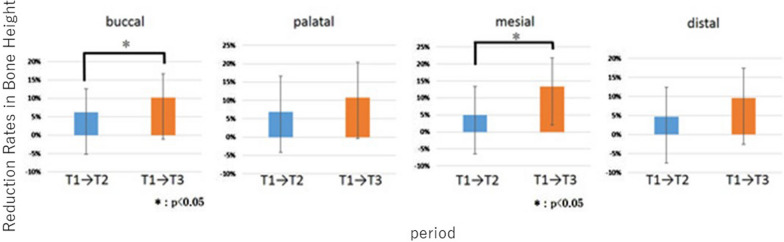
Reduction rates in bone height. T1, T2, and T3 indicate 1 year, 2 years, and 5 years after surgery, respectively

From T1 to T3, the average reduction rate in the surrounding bone height was 8% on the buccal side, 9% on the palatal, 12% on the mesial, and 4% on the distal. Bone height increased on the buccal side in two cases, palatal side in two, mesial side in three, and distal side in four. On the buccal and mesial sides, the average reduction rates in bone height from T1 to T3 were significantly higher than T1 to T2 (*p* = 0.036 and *p* = 0.033, respectively) (Fig. 8).

#### Intra-observer reliability

The value of intraclass correlation coefficient (ICC) for the augmented bone volume was 0.89. In addition, the ICC values for the reduction rate of the bone heights and the resorption rate of the TCP plate were also over 0.7. Conclusively, the results should be moderately reliable from a statistical viewpoint.

## Discussion

Autologous bone grafting was applied for the first case of bone augmentation for dental implants in the region of maxillary sinus [2]. Since then, large number of alternative bone substitutes have been studied due to excessive invasion caused by the collection of autologous bone grafts, and limitations in collected bone amounts. Allogeneic bones, xenograft bones, and alloplasts were validated in these studies, and bone tissue engineering procedures utilizing various cells and growth factors to obtain osteoinducibility were also evaluated [33–35]. These studies have been conducted considering that manipulation of osteogenic cells, growth factors, and osteoconductive scaffolds are important components of bone regenerative engineering. However, contrasting techniques wherein bone graft materials, cells, and growth factors were not used in sinus augmentation procedures have also been reported [36, 37]. In these techniques, blood supply is derived from the surface of the bone and the Schneiderian membrane after membrane elevation. In addition, growth factors present in the blood clot, and cells promoting bone formation are believed to be supplied from the surrounding bone and the Schneiderian membrane [38]. An animal study that verified osteoconduction of TCP blocks showed very strong osteoconduction from the calvarial bone, even when osteogenic cells and/or growth factors were not inserted [39]. Similarly, in this study, only blood clot was present in the space underneath the TCP plate, and histological findings confirmed new bone formation. We speculate that TCP with strong osteoconduction and the effect of growth factors from blood clot, along with osteoprogenitor cells from the surrounding tissues enabled sufficient bone formation. Methods of bone regeneration using tissue engineering may promote advanced and faster bone formation, however, it is not considered the best procedure in clinical practice due to the associated risks, cost factor and effort involved.

Several previous reports have verified bone gain and subsequent resorption after sinus augmentation, however, most of these studies used two-dimensional evaluation using panoramic radiographs [40–42]. Recent digitization has made it possible to use 3D bone models using DICOM data for planning implant treatments. Morphology of bone hyperplasia due to sinus augmentation is very complex, and insightful two-dimensional assessments are arduous. Several studies have reported evaluation of bone morphology after sinus augmentation using the 3D bone model used in implant treatment planning [28, 43, 44]. However, sinus augmentations reported till date for 3D evaluation were performed either using granular bone grafting material [45–47] or without any grafting material [37, 48, 49]. Although procedures using plate-shaped bone substitutes have been reported [50], no 3D evaluation of this technique using CBCT has been performed. In addition, no reports have shown a long-term prognosis of 5 years. Furthermore, to the best of our knowledge, this clinical study is the first to three dimensionally evaluate the resorption of block-shaped TCP alone in the maxillary sinus.

In addition, several of the reports till date have compared bone models, but in this study [28, 43, 44], the maxillary sinus was extracted and the volume was measured. At first, we also tried to measure using a bone model, but it was difficult to reproduce the bone model due to the thinness of the bone in the anterior wall of maxillary sinus and the influence of halation. Moreover, it was difficult to calculate using software as the amount of extracted data was too heavy. Thus, we tried to develop a method to assess bone volume after sinus augmentation and establish a novel method to measure the change in the augmented bone volume. Particularly, maxillary sinus extraction method in this study was not affected by bone thinness or X-ray halation as the range of extraction varied individually and the specific area was extracted appropriately. Therefore, this method is reliable and has high reproducibility. As described above, this novel digital measurement method can be used to quantify changes in bone volume after bone augmentation, especially sinus augmentation.

In the present study, the mean resorption rate of TCP plates after 2 years of surgery was 53%, however, this rate increased to 83% 5 years after surgery, with a confirmed resorption rate of 90% or more in six of nine cases. Artzir et al. reported that β-TCP particles resorbed completely and were replaced by newly formed bone within 24 months [51]. In this study, we hypothesized that complete resorption of TCP plates was prolonged due to the increased surface area and complex morphology caused by crystallization of calcium phosphate.

In this study, the reduction rate in bone volume was 15% after 2 years of surgery and 22% after 5 years. These reduction rates were considerably lesser than previous reports [52, 53]; which was probably because β-TCP plates resorbed more slowly, and the space was secured for a longer period of time. However, increased bone volumes were observed after surgery in two cases in this study. In these two cases, β-TCP plate resorption was considerably slower than the other cases and was replaced by newly formed bone 5 years after the surgery.

The average bone height reduction between 1–2 years and 1–5 years after surgery was 0.7 mm and 1.2 mm, respectively; and these values were lower than the previously reported values [54, 55]. In this study, significant differences in average bone height reduction were observed on the buccal and mesial sides, but not on the palatal or distal sides. To create the buccal window, the buccal bone was milled, and the sinus membrane was elevated. Consequently, the buccal bone disappeared, but the other side of the bony walls was maintained as shown in Fig. 1. Moreover, separation of the sinus membrane from the mesial bony wall and creation of a tension-free situation might be difficult. Therefore, buccal and mesial bone heights were significantly resolved during the post-operative 5 years as shown in Fig. 8. In one case, increase in bone height was observed at all sites. A previous clinical study showed that implant loading promoted osteogenesis over long term and exerted a stabilizing effect on the maintenance of bone graft height; which resulted in decrease in bone height during the first 2–3 years after surgery, and then increase in height until 96 months postoperatively [54]. In our study, all patients received their prostheses within 1 year of surgery. Therefore, we could assume that the decrease in bone height was relatively minuscule and the bone height was stable due to the mechanical force even after 5 years of surgery.

The β-TCP plate remained stable after surgery and maintained the bone height. The fact that all the subjects in this study were partially edentulous may also have affected the maintenance of bone height. However, as shown in Fig. 2, the area which was not supported by β-TCP plate appeared to collapse year by year. Five years after surgery, bone height was maintained even though the β-TCP plate had almost resorbed. However, collapse might occur in the future, so further observation is required.

This study had a few limitations. First, histological analysis was performed 6 months after sinus augmentation, but in only one case. As a result, immature new bone formation was observed, confirming the transformation of blood clots into mineralized tissue. Second, the sample size of this study was relatively small. However, a post hoc analysis, using the program G*power version 2 (Heinrich-Heine-Universität Düsseldorf, Düsseldorf, Germany) revealed that number of TCP plates for resorption rate analysis and bone reduction rate surrounding implants was enough and the results were reliable.

## Conclusions

This sinus augmentation technique provided an uneventful post-operative course for 5 years, and the use of a plate-shaped TCP demonstrated similar results as granular bone substitutes. Formation of new bone around the implant was confirmed, without the use of any bone graft material. Moreover, it was also observed that absorption of TCP took several years. These findings affirmed our hypothesis that the novel evaluation method with 3D quantification for augmented bone volume could successfully assess temporal changes in bone volume after sinus augmentation, and that plate-shaped TCP could provide higher security of augmented bone volume. Moreover, neither implant failure nor major complications were observed 5 years after the surgery, suggesting that this technique could result in long-term implant stability.

## Data Availability

Not applicable.

## Abbreviations

TCP: Tricalcium phosphate
3D: Three dimension
CBCT: Cone-beam computed tomography
STL: Standard triangulated language
HA: Hydroxyapatite
DICOM: Digital imaging and communications in medicine
CT: Computed tomography
